# Gestational Diabetes Mellitus: A Cross-Sectional Survey of Its Knowledge and Associated Factors among United Arab Emirates University Students

**DOI:** 10.3390/ijerph19148381

**Published:** 2022-07-08

**Authors:** Maryam M. Bashir, Luai A. Ahmed, Meera R. Alshamsi, Sara Almahrooqi, Taif Alyammahi, Shooq A. Alshehhi, Waad I. Alhammadi, Hind A. Alhosani, Fatima H. Alhammadi, Rami H. Al-Rifai, Fatma Al-Maskari

**Affiliations:** 1 Institute of Public Health, College of Medicine and Health Sciences, United Arab Emirates University, Al Ain P.O. Box 15551, United Arab Emirates; 201890105@uaeu.ac.ae (M.M.B.); luai.ahmed@uaeu.ac.ae (L.A.A.); 201600473@uaeu.ac.ae (M.R.A.); 201612377@uaeu.ac.ae (S.A.); 201605300@uaeu.ac.ae (T.A.); 201703784@uaeu.ac.ae (S.A.A.); 201813111@uaeu.ac.ae (W.I.A.); 201814146@uaeu.ac.ae (H.A.A.); 201706495@uaeu.ac.ae (F.H.A.); rrifai@uaeu.ac.ae (R.H.A.-R.); 2 Zayed Centre for Health Sciences, United Arab Emirates University, Al Ain P.O. Box 15551, United Arab Emirates

**Keywords:** gestational, diabetes, GDM, pregnancy, knowledge, students, United Arab Emirates

## Abstract

Gestational diabetes mellitus (GDM) burden is burgeoning globally. Correct knowledge about GDM among young people is paramount for timely prevention. This study assesses GDM knowledge and identifies factors associated with it among United Arab Emirates (UAE) University students. A validated self-administered questionnaire collected data from the university students. We analyzed the data for GDM knowledge status (ever heard of GDM) and GDM knowledge levels (poor, fair, and good) and conducted ordinal logistic regressions to assess for associated factors. A total of 735 students were surveyed with a mean age of 21.0 years. Of these, 72.8% had heard of GDM, and 52.9% of males versus 20.3% of female students had never heard of the condition before. Higher age (*p* = 0.019) and being a postgraduate student (*p* = 0.026) were associated with higher GDM knowledge status in males. GDM knowledge level analysis showed that 24.0%, 58.5%, and 17.5% had poor, fair, and good knowledge. The mean GDM-knowledge score was 6.3 ± 2.4 (out of 12). Being married [aOR-1.82 (95%CI 1.10–3.03)] and knowing someone who had GDM [aOR-1.78 (95%CI 1.23–2.60)] were independently associated with higher GDM knowledge levels among students. Students’ primary source of GDM knowledge was family/friends. There is an observed knowledge gap related to GDM among the students, especially males. This study urges the need to accelerate targeted GDM awareness campaigns among university students and the general population in the UAE.

## 1. Introduction

Gestational diabetes mellitus (GDM) is one of the classifications of hyperglycemia first detected during pregnancy which resolves at the end of the pregnancy [1,2]. It is one of the most common metabolic disorders during pregnancy, with short- and long-term consequences if undiagnosed or untreated. It leads to multiple perinatal complications in both mother and fetus [3,4,5,6,7]. There is also the future risk of developing type 2 diabetes mellitus (DM) among the mothers [8,9] and a long-term risk of developing obesity, hypertension, and type 2 DM among the babies of GDM mothers [10,11,12]. GDM is also associated with a higher risk of cardiovascular disorders and early vascular aging [13]. In addition, it has an overwhelming economic burden [14]. The global burden of GDM is increasing, mirroring the global epidemic of type 2 DM. This epidemic is even more evident in the Middle East and North Africa (MENA) region following rapid urbanization and obesity over the past few decades [9,15]. GDM prevalence in the MENA region ranges from 8.4% to 24.5% [16], with a pooled prevalence of 13.0% from a recent meta-analysis [17]. In the United Arab Emirates (UAE), GDM prevalence ranges from 7.9% to 24.9% [7,16,17], and in some cases, more than one in three pregnancies are affected by GDM [18].

Knowledge is essential for health literacy [19] and is linked to significant health outcomes [20]. Knowledge of GDM, especially its risk factors and complications, is essential to its management and preventive strategies, thereby reducing its burden [21,22]. Studies conducted worldwide to assess GDM knowledge have shown different results. A multicenter study among women attending antenatal care (ANC) in India showed that very few (6.3%) of the pregnant women were aware of GDM [23]. Conversely, Bhowmik and colleagues in 2018 in Bangladesh showed that 81.8% of their study participants were aware of GDM; however, the knowledge score (2.7 ± 1.5 out of 8) was low [21]. A United States study [24] among 85 pregnant women showed that none could correctly identify GDM risk factors. However, in a survey conducted among Polish women (pregnant and non-pregnant) in 2021, moderate GDM knowledge was noted where up to 47.5% of the women had intermediate GDM knowledge [25].

A large population study conducted in Saudi Arabia by Alharthi and colleagues showed the mean GDM knowledge score among participating women to be 5.5 ± 2.5 out of 12 [26]. In the UAE, a study conducted in 2017 among 450 women of childbearing age showed good awareness of GDM (73.5%); however, their knowledge levels were low [27]. Regarding associated factors, studies show that women who are older [24,28], have more pregnancies [23], and are employed [21] were significantly more aware and had a higher knowledge level of GDM. Ethnicity and educational status were also found to be predictors of GDM knowledge levels [28,29,30]. One study found that males had significantly higher knowledge scores than females [21]. The most popular source of GDM knowledge identified by studies is the internet. Other sources include family members or relatives, friends, school/workplace, hospital, parental books, magazines, and mass media [25,27,31,32,33].

Even though several studies have assessed GDM knowledge worldwide, most studies were conducted among pregnant women. Very few assessed GDM knowledge among the general population, and even fewer studies included males. Fathers’ involvement in maternal care has been found to increase early ANC visits and positively affect both maternal and child health [34]. Furthermore, knowing a population’s GDM knowledge level is essential for implementing preventive strategies. However, there is currently a paucity of such data in the UAE, and to our knowledge, this is the first study conducted among university students in the Middle East and North Africa (MENA) region to assess their GDM knowledge. The present study aimed to investigate the gestational diabetes mellitus (GDM) knowledge status, level, and source of knowledge among university students and to identify factors associated with these.

## 2. Materials and Methods

### 2.1. Study Design and Study Setting

We conducted a cross-sectional study among United Arab Emirates University (UAEU) students between July and October 2021 to assess their overall status and levels of GDM knowledge. More than 13,000 graduate and undergraduate students are currently enrolled in the University.

### 2.2. Participants and Sampling

All students from 8 out of 9 colleges, males and females, undergraduates, and postgraduates, who were 18 years and above and gave their informed consent, were eligible for the study. All students in UAEU were invited to join the survey via their school emails. Those who responded and fulfilled the eligibility criteria were included in the study. The College of Medicine and Health Sciences (CMHS) was excluded from this study as its students’ level and source of knowledge would impact the overall results.

### 2.3. Sample Size

The sample size was calculated using online OpenEpi Version 3.01. A minimum sample of 360 participants will allow the detection of a true proportion (73.5%) of those who have ever heard of GDM before [27], given 80% power, 5% alpha error, and accounting for 20% non-response.

### 2.4. Data Collection and Procedure

Data collection was performed via an online self-administered questionnaire using the SurveyMonkey software. A link to the survey was shared with students through emails by the deaneries of all colleges. The link contained study details, informed consent forms, and questionnaires (Arabic and English versions) (Supplementary Figure S1). The survey link was an anonymous link generated by SurveyMonkey. Participating students filled out the consent forms and questionnaires online, and their data were securely downloaded and accessed by only the researchers. No identifier variable was collected, making the data collection completely anonymous. Students were assured of complete anonymity in the informed consent form.

We adapted our questionnaire from the Arabic and English questionnaires validated by Alharthi and colleagues in 2018 among 9002 members of the Saudi Arabian population [26]. Our questionnaire was divided into three sections. The first section collected information on students’ sociodemographic variables, the second assessed GDM knowledge levels, and the third assessed sources of knowledge.

Section 1 contained 20 questions, including students’ sociodemographic characteristics and a question inquiring whether the students have ever heard of GDM before (GDM knowledge status). Participants who answered “No” were automatically taken to the end of the survey, while those who answered “Yes” were taken to the second section.

Section 2 contained 12 questions inquiring about several aspects of GDM. Five questions were asked about GDM risk factors, two on diagnosis, two on management, and three on GDM-related complications. Each question was given 1 point if answered correctly. In the questionnaire, there were nine main questions in this section, and questions 8 and 9 were multiple-choice, carrying 2 and 3 points, respectively (See Supplementary Figure S1). The potential GDM-knowledge score could range from 0 to 12. Technical terms were avoided as much as possible; descriptions of terms were given instead. For example, OGTT was described as “Blood analysis after drinking glucose solution.”

Section 3 inquired about sources of GDM knowledge. The potential sources of the knowledge were hospitals, colleges, workplaces, TV, newspapers/magazines, social media, other online resources, family/friends, neighborhoods, and others. The questionnaire was pre-tested among 50 students (25 Arabic and 25 English versions) and assessed for clarity, comprehension, and order of questions.

### 2.5. Outcome Variables

We had two main outcomes. The first outcome was a binary variable showing the GDM-knowledge status among the surveyed students. This outcome was categorized as either “ever heard of GDM” or “never heard of GDM.”

The second outcome showed GDM knowledge levels among students who reported having heard of GDM. This outcome was categorized based on the GDM knowledge points as poor knowledge (≤4), fair knowledge (5–8), and good knowledge (9–12) [26]. We also summarized the total GDM knowledge points or score (out of 12) as a continuous variable.

### 2.6. Associated Factors

We assessed students’ sociodemographic characteristics, including age, gender, program (undergraduate or postgraduate), marital status, working status, having someone at home working in the health sector, knowing someone who had GDM, family history of diabetes, self-reported weight, and height.

### 2.7. Statistical Analysis

We conducted all analyses using STATA statistical software version 16.1 (StataCorp LLC, College Station, TX, USA). Students’ characteristics and GDM knowledge were summarized using appropriate descriptive statistics.

We assessed the proportions of students who had “ever heard of GDM” and “never heard of GDM” (GDM-knowledge status) and compared students’ characteristics between the two groups. Continuous variables were compared using a *t*-test or Wilcoxon rank-sum (Mann–Whitney U) test, while categorical variables were compared using a Chi-square test. The comparisons were made separately also for male and female students. Logistic regression was also conducted to assess associations (reported in Supplementary Materials).

The proportions of students with poor, fair, and good GDM knowledge (GDM knowledge levels) were also assessed, and students’ characteristics were compared among these groups using the Kruskal–Wallis test for continuous variables and Chi-square test for categorical variables. Simple and multiple ordinal logistic regression models were used to assess the associations between students’ characteristics and their GDM knowledge levels, fitting the statistically significant variables in the univariate models into the multiple regression model.

Regression results were expressed as adjusted odds ratio (aOR) with a 95% confidence interval (CI). The level of significance was specified at 5%. The normality of data was tested using the Shapiro–Wilk test. Missing data were addressed using complete case analysis because of minimal missing data (Supplementary Table S1).

## 3. Results

A total of 938 students responded to the survey. We excluded non-eligible students; hence, we had 735 eligible students (See Supplementary Figure S2).

### 3.1. Students’ Sociodemographic Characteristics

The median age of all the students (735) was 21.0 (Interquartile Range (IQR) 3.0) years. Most of the students were females (78.9%), single (85.6%), and undergraduates (82.3%). Of the 735 students, 50.2% had a family history of type 2 diabetes mellitus (DM), and 19.1% had someone in their homes working in the health sector. 

Out of the 735 eligible students, 72.8% had heard of GDM, while 27.2% had never heard of GDM before. Gender is independently associated with ever hearing of GDM in this study population (Female vs. Male, 79.7 vs. 47.1, respectively, *p* < 0.001, aOR (95%CI) − 3.444 (1.944–6.102)) (Supplementary Tables S2 and S4). Table 1 and Table 2 describe the sociodemographic characteristics among female and male students, respectively, and their associations with GDM knowledge status.

### 3.2. Students’ GDM Knowledge Status

Among 580 female students, 79.7% had heard of GDM before. The characteristics assessed were not significantly associated with the female students’ GDM knowledge status (Table 1).

On the other hand, less than half (47.1%) of the male students had heard of GDM. Increased age (*p* = 0.019) was associated with their GDM knowledge status, and more postgraduate male students had heard of the condition than their undergraduate counterparts (60.9% vs. 41.3%, respectively, *p* = 0.026). The other male students’ characteristics were not significantly associated with their GDM knowledge status (Table 2).

### 3.3. Students’ GDM Knowledge Levels

Out of the 735 eligible students, 200 had never heard of GDM and were automatically excluded from answering Section 2 of the questionnaire on GDM knowledge levels. Of the 535 who had heard of GDM before, 55 students did not complete Section 2 and were excluded from the GDM knowledge levels analysis too. Hence, 480 participating students were included in the knowledge levels analysis (Supplementary Figure S2).

Table 3 shows GDM knowledge levels among the 480 students and the factors associated with the levels. 24.0% of these students had poor knowledge of GDM, 58.5% had fair knowledge, and 17.5% had good knowledge. The total median GDM knowledge points out of 12.0 among the 480 students was 6.0 (IQR 3.0) [mean = 6.3 ± 2.4]. The medians among those with poor, fair, and good knowledge were 4.0 (IQR 2.0), 7.0 (IQR 1.0), and 10.0 (IQR 1.0), respectively (*p* < 0.001). Being married and knowing someone who had GDM were significantly associated with GDM knowledge levels. In contrast, the other compared characteristics were not.

### 3.4. Regression Analysis for GDM Knowledge levels

Simple ordinal logistic regression was performed using age, gender, program (undergraduate or postgraduate), marital status, working status, having someone at home working in the health Sector, knowing someone who had GDM, family history of diabetes, and BMI. Only marital status and knowing someone who had GDM were found to be significant and were included in the adjusted model. Following multiple ordinal logistic regression, results showed that the odds of having a higher GDM knowledge level among students who were married increased by 82% compared to single students [aOR 1.82 (95% CI 1.10–3.03)]. Students who knew someone with GDM had 78% higher odds of having good GDM knowledge compared to those who did not [aOR 1.78 (95% CI 1.23–2.60)] (Table 4).

### 3.5. Knowledge Domains (GDM Risk Factors, Diagnosis, Management, and Complications)

**Figure 1:**
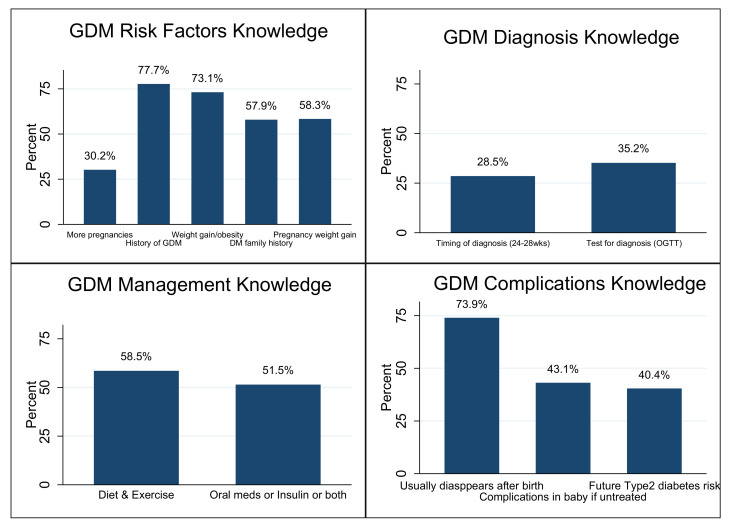
This figure shows participating students’ knowledge of gestational diabetes mellitus (GDM) risk factors, diagnosis, management, and complications (N = 480).

Out of the five GDM risk factors assessed, personal history of GDM (77.7%) and pre-pregnancy weight gain/obesity (73.1%) were the most identified by the 480 students, while having more pregnancies (30.2%) was the least identified. A total of 35.2% of the students correctly identified the test used for GDM diagnosis, 28.5% correctly identified the timing and 58.5% knew that diet and exercise are part of GDM management. Meanwhile, only 43.1% and 40.4% of these students knew that GDM leads to baby complications and increased future risk of type 2 DM, respectively (Figure 1).

Overall, only 5 (1.04%) students could answer all 12 points correctly, 7 (1.46%) could answer none of the 12 points correctly, and 5 (1.04%) could correctly answer at least one point (out of 12) (Supplementary Table S3).

### 3.6. Sources of GDM Knowledge among the Students

**Figure 2:**
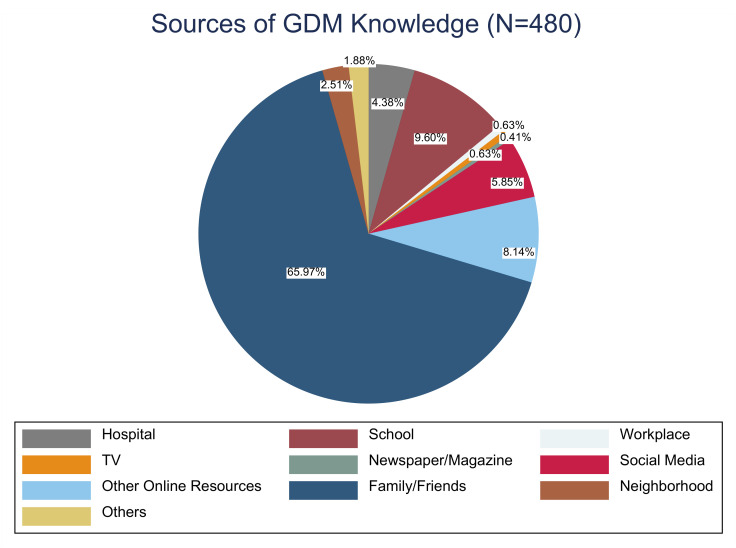
This figure shows the sources of GDM knowledge in the study population.

Of the ten sources of GDM knowledge assessed among the 480 students, the most popular were family/friends (65.9%), school (9.6%), other online resources (8.1%), and social media (5.9%). The least popular sources were TV (0.6%) and newspapers/magazines (0.4%) (Figure 2).

## 4. Discussion

This study, conducted among 735 university students in the UAE to assess their GDM knowledge status and levels, showed that almost three-quarters were aware of GDM, i.e., had heard of GDM before. Gender was identified to be independently associated with this knowledge status. More than half of the male students had never heard of the condition before, and higher age and being a postgraduate student were significantly associated with higher GDM knowledge among them. The study also showed that knowledge levels were mostly fair among students who had heard of the condition. Marital status and knowing someone who had GDM were independent factors associated with students’ knowledge levels. GDM knowledge sources were mainly friends/families, school, and the internet.

Despite our study population’s overall good GDM knowledge status, there is a significant knowledge gap among the students, especially since GDM prevalence is high locally and regionally [16,18]. GDM knowledge status in our study population is similar to the study conducted among adult women in Sharjah, where 73.5% have heard about GDM before [27]. However, it is lower than the Bangladeshi study, where 81.8% of participants were aware of GDM [21], and higher than in many studies conducted in different parts of India, Nigeria, Samoa, and Uganda [23,28,32,33,35,36]. Most of these studies were conducted among pregnant women, who were expected to be more aware of the condition than our study population.

The GDM knowledge level among our study population was fair, though about one in four students had poor knowledge. Knowledge levels in our study are similar to those found in the studies in India [33] and Norway [30], and to some extent, in the Saudi Arabia study [26] where 54.8% of the women had fair GDM knowledge, though more participants (33.8%) had poor knowledge than our study population. This proportion is also similar to the Polish study [25]. Compared to the other UAE study in Sharjah [27], the GDM knowledge level among our study population is higher.

Our study has highlighted the difference in GDM knowledge status between male and female students. More than half of the male students who participated were unaware of the condition; hence, female students were likelier to have heard of GDM. Males younger and at the undergraduate level were even less likely to have heard of it. For GDM knowledge levels (good, fair, or poor), significant differences according to gender were not detected, unlike the Bangladesh study [21], where men had significantly higher knowledge levels than women. Significant associated factors of knowledge levels in this study (after multiple regression) include being married and knowing someone who had GDM. Many studies [23,24,28,37] highlighted age and educational status as predictors of GDM knowledge levels, but this is not the case in our study. The Saudi study [26], on the other hand, supports our study’s finding that knowing someone who had GDM is positively associated with knowledge levels.

Looking at the knowledge domains, more students had higher knowledge of GDM risk factors than its diagnosis, management, and complications. These risk factors are some of the most commonly identified in the UAE population [38,39,40]. Less than half of the students knew about GDM diagnosis (timing and test type), and correct knowledge could encourage early ANC presentation and GDM screening. Knowledge of GDM diagnosis is also not widespread in other populations [33]. GDM management knowledge among participants was average, though almost half of the students did not know that diet and exercise are part of GDM management. This is one of the most important things to know about GDM to promote proactive healthy lifestyle changes. Most students were also unaware of baby complications and the risk of type 2 DM in the future if untreated. The students’ knowledge of these domains is higher than in the US study among ANC women [24]. Our study participants had a median GDM knowledge score of 6 out of 12 and a mean of 6.3, which is higher than the Saudi study [26], from where we adapted our assessment tool.

The primary source of GDM knowledge among the students was family or friends. This finding is supported by the Sharjah study [27], among others [32,36]. This likely supports our finding that knowing someone with GDM increases the chances of having a deeper knowledge of it, and may partially explain why more than half of the males in this population had never heard of GDM before. Culturally, in this setting, women are more likely to discuss pregnancy issues with other women among friends and families than men. This highlights the need for more and broader community-based awareness campaigns to disseminate correct GDM knowledge to the population. The internet (social media and other resources), also popular among students, could be used as one of the platforms for awareness campaigns among them. As expected, mass media is not a popular source among our study population.

One of the strengths of our study is the large sample size which increases the precision of estimates by reducing margins of error [41]. To our knowledge, this is the first study to report GDM knowledge among university students in the MENA region. And it is also the first study in the region to report GDM knowledge among males. Hence, our study covers an essential gap on this topic as most studies were conducted among pregnant women. The main limitation includes not being able to use a random sampling technique. This might affect the representativeness of our sample. Nevertheless, we made efforts to ensure more comprehensive recruitment by reminding the deans to send group email reminders with the survey links weekly to the students in different departments of their colleges. There is also an issue of subjectivity related to the self-reported data, especially since the questionnaires were filled out online. Students were requested at the beginning of the questionnaire to answer the questions using their current knowledge. In this study, we did not assess the students’ socioeconomic backgrounds in relation to their GDM knowledge, as the effect of different socioeconomic statuses was linked to individuals’ educational levels [21]. We recommend assessing the socioeconomic status in future similar studies among the general population.

## 5. Conclusions

Our study showed almost three-quarters of the university students had heard of GDM; hence, about one-quarter had never heard of it before. It highlights the need for GDM awareness campaigns among university students, especially among the identified groups with knowledge gaps. In addition, there is a need to involve the general population, as we found it to be the primary source of students’ GDM knowledge. GDM awareness should be included in preconception care, and male involvement should be encouraged. Premarital counseling and screening programs could be a golden opportunity to initiate GDM awareness and preventive strategies, especially among young adults considering the knowledge gap. More research should be conducted to assess such programs’ short- and long-term effectiveness.

## Figures and Tables

**Table 1:** Sociodemographic characteristics of female students and factors associated with their GDM knowledge status (N = 580).

Sociodemographic Characteristics	Total Population N *	n (%)/Median (IQR)	Knowledge Status		*p*-Value ^a^
			Ever-Heard of GDM n = 462 (79.7%)	Never-Heard of GDM n = 118 (20.3%)	
**Age (years)**	580	20.0 (4.0)	20.0 (4.0)	20.0 (4.0)	0.483
**Weight (kg)**	554	58.0 (17.0)	58.0 (18.0)	58.5 (16.0)	0.553
**Height (m)**	558	1.6 (0.1)	1.6 (0.1)	1.6 (0.1)	0.592
**BMI (kg/m^2^)**	551	22.9 (6.1)	22.9 (6.4)	22.5 (5.3)	0.409
**Program**	580				
**Undergraduate**		496 (85.5)	396 (79.8)	100 (20.2)	
**Postgraduate**		84 (14.5)	66 (78.6)	18 (21.4)	0.071
**Marital status**	580				
**Single**		506 (87.2)	397 (78.5)	109 (21.5)	
**Married**		74 (12.8)	65 (87.8)	9 (12.2)	0.061
**Working status**	580				
**Yes**		50 (8.6)	39 (78.0)	11 (22.0)	
**No**		530 (91.4)	423 (79.8)	107 (20.2)	0.093
**Anyone in your home working in the Health Sector**	580				
**Yes**		111 (19.1)	85 (76.6)	26 (23.4)	
**No**		469 (80.9)	377 (80.4)	92 (19.6)	0.370
**Family history of diabetes**	580				
**Yes**		303 (52.2)	246 (81.2)	57 (18.8)	
**No**		277 (47.8)	216 (78.0)	61 (22.0)	0.337

Data were expressed as median (IQR—Interquartile range) or n (%) unless stated otherwise. * = Total number of students who responded to a particular question. ^a^ = Chi square test was used for categorical variables and the Mann–Whitney U test for continuous variables.

**Table 2:** Sociodemographic characteristics of male students and factors associated with their GDM knowledge status (N = 155).

Sociodemographic Characteristics	Total Population N *	n (%)/Median (IQR)	Knowledge Status		*p*-Value ^a^
			Ever Heard of GDM n = 73 (47.1%)	Never Heard of GDM n = 82 (52.9%)	
**Age (years)**	155	21.0 (7.0)	22.0 (8.0)	21.0 (4.0)	0.019
**Weight (kg) Mean (SD)**	144	77.2 (16.2)	78.3 (16.4)	76.3 (16.0)	0.464 ^b^
**Height (m)**	126	1.7 (0.1)	1.7 (0.1)	1.7 (0.1)	0.136
**BMI (kg/m^2^)**	126	25.4 (6.5)	25.5 (7.0)	25.2 (5.8)	0.254
**Program**	155				
**Undergraduate**		109 (70.3)	45 (41.3)	64 (58.7)	
**Postgraduate**		46 (26.7)	28 (60.9)	18 (39.1)	0.026
**Marital status**	155				
**Single**		123 (79.4)	53 (43.1)	70 (56.9)	
**Married**		32 (20.6)	20 (62.5)	12 (37.5)	0.050
**Working status**	155				
**Yes**		39 (25.2)	22 (56.4)	17 (43.6)	
**No**		116 (74.8)	51 (44.0)	65 (56.0)	0.178
**Anyone in your home working in the Health Sector**	155				
**Yes**		29 (18.7)	15 (51.7)	14 (48.3)	
**No**		126 (81.3)	58 (46.0)	68 (54.0)	0.580
**Family history of diabetes**	155				
**Yes**		66 (42.6)	37 (56.1)	29 (43.9)	
**No**		89 (57.4)	36 (40.5)	53 (59.5)	0.054

Data were expressed as median (IQR—Interquartile range) or n (%) unless stated otherwise. * = Total number of students who responded to a particular question. ^a^ = Chi square test was used for categorical variables, while t-test ^b^ and Mann–Whitney U test were used for continuous variables.

**Table 3:** Descriptive statistics showing factors associated with GDM knowledge levels (poor, fair, and good knowledge) among participating students (N = 480).

			Knowledge Levels			
Students’ Characteristics	Total Population N *	%/Median (IQR)	Poor GDM Knowledge ^b^ 115 (24.0%) %	Fair GDM Knowledge ^b^ 281 (58.5%) %	Good GDM Knowledge ^b^ 161 (17.5%) %	*p*-Value ^a^
**Age (years)**	480	21.0 (3.0)	21.0 (3.0)	21.0 (4.0)	21.0 (5.0)	0.170
**Weight (kg)**	457	60.0 (21.0)	60.0 (20.0)	59.0 (21.0)	65.0 (20.0)	0.185
**Height (m)**	452	1.6 (0.1)	1.6 (0.1)	1.6 (0.1)	1.6 (0.1)	0.440
**BMI (kg/m^2^)**	449	23.3 (6.9)	23.1 (7.1)	23.1 (7.0)	24.4 (6.4)	0.384
**Gender**	480					
**Male**		13.8	31.8	56.1	12.1	
**Female**		86.2	22.7	58.9	18.4	0.190
**Program**	480					
**Undergraduate**		81.7	24.2	59.7	16.1	
**Postgraduate**		18.3	22.7	53.4	23.9	0.218
**Marital status**	480					
**Single**		84.6	24.4	60.8	14.8	
**Married**		15.4	21.6	46.0	32.4	0.001
**Working status**	480					
**Yes**		11.7	23.2	55.4	21.4	
**No**		88.3	24.1	58.9	17.0	0.710
**Anyone in your home working in the Health Sector**	480					
**Yes**		19.0	18.7	57.1	24.2	
**No**		81.0	25.2	58.9	15.9	0.122
**Family history of diabetes**	480					
**Yes**		53.8	20.9	62.0	17.1	
**No**		46.2	27.5	54.5	18.0	0.187
**Know someone who had GDM**	480					
**Yes**		65.6	20.3	59.1	20.6	
**No**		34.4	30.9	57.6	11.5	0.006
**Total GDM knowledge points (out of 12)**	480	6.0 (3.0)	4.0 (2.0)	7.0 (1.0)	10.0 (1.0)	<0.001

Data were expressed as median (IQR- Interquartile range) or percentages (%) unless stated otherwise. * = the Total number of students who responded to a particular question. ^a^ = Chi square used for categorical variables and Kruskal–Wallis test for continuous variables. ^b^ = poor knowledge (≤4), fair knowledge (5–8), and good knowledge (9–12).

**Table 4:** Multiple regression analysis showing factors associated with GDM knowledge levels (poor, fair, good) among students (N = 480).

Students’ Characteristics		Adjusted OR (95% CI) *
**Marital status**	Single	ref
	Married	1.82 (1.10–3.03) ^a^
**Know someone who had GDM**	No	ref
	Yes	1.78 (1.23–2.60) ^a^

* = Ordinal logistic regression used. ^a^ = Statistically significant (*p* < 0.05).

## Data Availability

Study data are available upon request from the corresponding author, subject to approval by UAEU Social Sciences Research Ethics Committee.

## Supplementary Materials

The following supporting information can be downloaded at: https://www.mdpi.com/article/10.3390/ijerph19148381/s1. Supplementary Table S1: Supplementary table showing the percentage of missing data for the 735 eligible students; Supplementary Table S2: Sociodemographic characteristics of 735 eligible students; Supplementary Table S3: Supplementary Table showing the frequency of GDM Knowledge points (0–12) among study population (480 Students); Supplementary Table S4: Supplementary Table showing detailed results of simple and multiple regression analysis between GDM knowledge status (ever heard of GDM and never heard of GDM) and students’ characteristics (N = 735); Supplementary Figure S1: Questionnaires (Arabic and English versions); Supplementary Figure S2: Supplementary Figure showing study population.

## Abbreviations

GDM	gestational diabetes mellitus
DM	diabetes mellitus
ANC	antenatal care
OGTT	oral glucose tolerance test
BMI	body mass index
UAE	United Arab Emirates

## References

[1] (2014). Diagnosis and classification of diabetes mellitus. Diabetes Care.

[2] (2014). Diagnostic criteria and classification of hyperglycaemia first detected in pregnancy: A World Health Organization Guideline. Diabetes Res. Clin. Pract..

[3] Stacey T., Tennant P., McCowan L., Mitchell E., Budd J., Li M., Thompson J., Martin B., Roberts D., Heazell A. (2019). Gestational diabetes and the risk of late stillbirth: A case–control study from England, UK. BJOG Int. J. Obstet. Gynaecol..

[4] The Lancet (2019). Gestational diabetes in England: Cause for concern. Lancet.

[5] Coustan D.R., Lowe L.P., Metzger B.E., Dyer A.R., International Association of Diabetes and Pregnancy Study Groups (2010). The Hyperglycemia and Adverse Pregnancy Outcome (HAPO) study: Paving the way for new diagnostic criteria for gestational diabetes mellitus. Am. J. Obs. Gynecol..

[6] Garrison A. (2015). Screening, diagnosis, and management of gestational diabetes mellitus. Am. Fam. Physician.

[7] Agarwal M.M., Dhatt G.S., Punnose J., Koster G. (2005). Gestational diabetes: Dilemma caused by multiple international diagnostic criteria. Diabet. Med..

[8] Kim C., Newton K.M., Knopp R.H. (2002). Gestational diabetes and the incidence of type 2 diabetes: A systematic review. Diabetes Care.

[9] Agarwal M.M. (2020). Gestational Diabetes in the Arab Gulf Countries: Sitting on a Land-Mine. Int. J. Environ. Res. Public Health.

[10] Fetita L.S., Sobngwi E., Serradas P., Calvo F., Gautier J.F. (2006). Consequences of fetal exposure to maternal diabetes in offspring. J. Clin. Endocrinol. Metab..

[11] Saravanan P., Magee L.A., Banerjee A., Coleman M.A., Von Dadelszen P., Denison F., Farmer A., Finer S., Fox-Rushby J., Holt R. (2020). Gestational diabetes: Opportunities for improving maternal and child health. Lancet Diabetes Endocrinol..

[12] Lu J., Zhang S., Li W., Leng J., Wang L., Liu H., Li W., Zhang C., Qi L., Tuomilehto J. (2019). Maternal Gestational Diabetes Is Associated With Offspring’s Hypertension. Am. J. Hypertens..

[13] Seeland U., Nemcsik J., Lønnebakken M.T., Kublickiene K., Schluchter H., Park C., Pucci G., Mozos I., Bruno R.M. (2021). Sex and Gender Aspects in Vascular Ageing—Focus on Epidemiology, Pathophysiology, and Outcomes. Heart Lung Circ..

[14] Dall T.M., Yang W., Gillespie K., Mocarski M., Byrne E., Cintina I., Beronja K., Semilla A.P., Iacobucci W., Hogan P.F. (2019). The Economic Burden of Elevated Blood Glucose Levels in 2017: Diagnosed and Undiagnosed Diabetes, Gestational Diabetes Mellitus, and Prediabetes. Diabetes Care.

[15] Alawadi F., Abusnana S., Afandi B., Aldahmani K.M., Alhajeri O., Aljaberi K., Alkaabi J., Almadani A., Bashier A., Beshyah S.A. (2020). Emirates Diabetes Society Consensus Guidelines for the Management of Type 2 Diabetes Mellitus—2020. Dubai Diabetes Endocrinol. J..

[16] Zhu Y., Zhang C. (2016). Prevalence of Gestational Diabetes and Risk of Progression to Type 2 Diabetes: A Global Perspective. Curr. Diab. Rep..

[17] Al-Rifai R.H., Abdo N.M., Paulo M.S., Saha S., Ahmed L.A. (2021). Prevalence of Gestational Diabetes Mellitus in the Middle East and North Africa, 2000-2019: A Systematic Review, Meta-Analysis, and Meta-Regression. Front. Endocrinol..

[18] Agarwal M.M., Dhatt G.S., Shah S.M. (2010). Gestational diabetes mellitus: Simplifying the international association of diabetes and pregnancy diagnostic algorithm using fasting plasma glucose. Diabetes Care.

[19] Baker D.W. (2006). The meaning and the measure of health literacy. J. Gen. Intern. Med..

[20] Cavanaugh K.L. (2011). Health literacy in diabetes care: Explanation, evidence and equipment. Diabetes Manag..

[21] Bhowmik B., Afsana F., Ahmed T., Siddiquee T., Ahmed T., Pathan F., Mahtab H., Khan A.K.A. (2018). Evaluation of knowledge regarding gestational diabetes mellitus: A Bangladeshi study. Public Health.

[22] Draffin C.R., Alderdice F.A., McCance D.R., Maresh M., Harper Md Consultant Physician R., McSorley O., Holmes V.A. (2016). Exploring the needs, concerns and knowledge of women diagnosed with gestational diabetes: A qualitative study. Midwifery.

[23] Thomas S., Pienyu R., Rajan S.K. (2020). Awareness and Knowledge About Gestational Diabetes Mellitus Among Antenatal Women. Psychol. Community Health.

[24] Gastrich M., Peck S., Janevic T., Bachmann G., Lotwala N., Siyam A. (2013). Gestational diabetes mellitus: An educational opportunity Article points. J. Diabetes Nurs..

[25] Lis-Kuberka J., Orczyk-Pawiłowicz M. (2021). Polish Women Have Moderate Knowledge of Gestational Diabetes Mellitus and Breastfeeding Benefits. Int. J. Environ. Res Public Health.

[26] Alharthi A.S., Althobaiti K.A., Alswat K.A. (2018). Gestational Diabetes Mellitus Knowledge Assessment among Saudi Women. Open Access Maced. J. Med. Sci..

[27] Elmekresh A. (2017). Gestational diabetes awareness in women of childbearing age in Sharjah. Glob. J. Obes. Diabetes Metab. Syndr..

[28] Byakwaga E., Sekikubo M., Nakimuli A. (2021). Level of and factors associated with awareness of gestational diabetes mellitus among pregnant women attending antenatal care at Kawempe National Referral Hospital: A cross sectional study. BMC Pregnancy Childbirth.

[29] Carolan M., Steele C., Margetts H. (2010). Knowledge of gestational diabetes among a multi-ethnic cohort in Australia. Midwifery.

[30] Borgen I., Garnweidner-Holme L.M., Jacobsen A.F., Fayyad S., Cvancarova Småstuen M., Lukasse M. (2019). Knowledge of gestational diabetes mellitus at first consultation in a multi-ethnic pregnant population in the Oslo region, Norway—A cross-sectional study. Ethn. Health.

[31] Salhi A.A., Intern M., Alshahrani M.S., Alyamin M.M., Hamdi W.A., Alyami S.R., Almagbool A.S., Almoqati N.H., Almoqati S.H., Al-Saaed E.A.A.N. (2019). Assessment of the knowledge of pregnant women regarding the effects of GDM on mothers and neonates at a Maternal and Children hospital in Najran, Saudi Arabia. IJMDC.

[32] Ogu R.N., Maduka O., Agala V., Alamina F., Adebiyi O., Edewor U., Porbeni I., Abam C. (2020). Gestational Diabetes Mellitus Knowledge Among Women of Reproductive Age in Southern Nigeria: Implications for Diabetes Education. Int. Q. Community Health Educ..

[33] Shriraam V., Rani M.A., Sathiyasekaran B.W., Mahadevan S. (2013). Awareness of gestational diabetes mellitus among antenatal women in a primary health center in South India. Indian J. Endocrinol. Metab..

[34] Martin L.T., McNamara M.J., Milot A.S., Halle T., Hair E.C. (2007). The effects of father involvement during pregnancy on receipt of prenatal care and maternal smoking. Matern. Child Health J..

[35] Bhavadharini B., Deepa M., Nallaperumal S., Anjana R., Mohan V. (2017). Knowledge about gestational diabetes mellitus amongst pregnant women in South Tamil Nadu. J. Diabetol..

[36] Price L.A., Lock L.J., Archer L.E., Ahmed Z. (2017). Awareness of Gestational Diabetes and its Risk Factors among Pregnant Women in Samoa. Hawaii J. Med. Public Health.

[37] Hussain Z., Yusoff Z.M., Sulaiman S.A. (2015). Evaluation of knowledge regarding gestational diabetes mellitus and its association with glycaemic level: A Malaysian study. Prim. Care Diabetes.

[38] Abdulrahman M., Tabatabaei Z., Maqbool S., Hafidh K., Husain Z.S., Al Raeesi F.H., Abo Sada N.M., Akbar M., Hubaishi N.M., Tahlak M.A.R. (2020). A review of gestational diabetes mellitus management, risk factors, maternal and neonatal outcomes in two major maternity hospitals in the United Arab Emirates: A report from Dubai. J. Neonatal. Perinat. Med..

[39] Alkaabi J., Almazrouei R., Zoubeidi T., Alkaabi F.M., Alkendi F.R., Almiri A.E., Sharma C., Souid A.-K., Ali N., Ahmed L.A. (2020). Burden, associated risk factors and adverse outcomes of gestational diabetes mellitus in twin pregnancies in Al Ain, UAE. BMC Pregnancy Childbirth.

[40] Hashim M., Radwan H., Hasan H., Obaid R.S., Al Ghazal H., Al Hilali M., Rayess R., Chehayber N., Mohamed H.J.J., Naja F. (2019). Gestational weight gain and gestational diabetes among Emirati and Arab women in the United Arab Emirates: Results from the MISC cohort. BMC Pregnancy Childbirth.

[41] Roessner V. (2014). Large sample size in child and adolescent psychiatric research: The way of salvation?. Eur. Child Adolesc. Psychiatry.

