# Supplementary material for: Gestational Diabetes Mellitus: A Cross-Sectional Survey of Its Knowledge and Associated Factors among United Arab Emirates University Students
Source: Int J Environ Res Public Health. 2022 Jul 8;19(14):8381. doi: 10.3390/ijerph19148381 (PMC9321366; doi:10.3390/ijerph19148381)
Supplement: Supplementary file 1 [file ijerph-19-08381-s001.zip › Supplementary Figure S1 - Questionnaire (Arabic and English) GDM knowledge study.pdf]

Assessment of GDM Knowledge and Associated Factors among University Students in the UAE – A Cross-Sectional Study

Dear Respondent,
Please kindly fill the survey after signing the informed consent above. Thank you for your valuable time.
* Required

SECTION 1: SOCIODEMOGRAPHIC CHARCTERISTICS

Age*	_________
Gender*	☐ Female<br>☐ Male
Emirate*	☐ Abu Dhabi<br>☐ Dubai<br>☐ Sharjah<br>☐ Ajman<br>☐ Ras Al Khaimah<br>☐ Umm Al Quwain<br>☐ Fujairah
City*	☐ _________
Weight	☐ _________
Height	☐ _________
Marital status*	☐ Single<br>☐ Married<br>☐ Divorced<br>☐ Widow<br>☐ Other _________
Have you (or your spouse) been pregnant before? *(if applicable)*<br>If yes, how many pregnancies?	☐ Yes _________<br>☐ No<br>☐ NA
College*	☐ College of Business and Economics<br>☐ College of Humanities and Social Science<br>☐ College of Information Technology<br>☐ College of Engineering<br>☐ College of Education<br>☐ College of Law<br>☐ College of Agricultural and Veterinary Medicine<br>☐ College of Science<br>☐ College of Medicine and Health Science
Department*	☐ _________
Program*	☐ Undergraduate<br>☐ Post graduate
Are you currently working as well? *	☐ Yes<br>☐ No
If you are currently working, where	☐ I work in the health sector

do you work?	☐ I work outside the health sector<br>☐ NA
Is there anyone in your home working in the health field? *	☐ Yes ________<br>☐ No
Does anyone in your family have diabetes? Specify *	☐ Yes<br>☐ No
Do you have any chronic diseases?	a. Yes<br>b. No
If yes specify. (You can choose more than one answer)	☐ Pre-diabetes<br>☐ diabetes mellitus,<br>☐ hypertension,<br>☐ lipid/cholesterol abnormalities,<br>☐ heart disease,<br>☐ stroke,<br>☐ thyroid disease,<br>☐ obesity,<br>☐ others ____
Before now, have you ever heard what is called (gestational diabetes)? *	☐ Yes<br>☐ No
Do you know someone who has had gestational diabetes? *	☐ Yes<br>☐ No<br>☐ NA
Did you (or your spouse) have gestational diabetes during any of your pregnancies? *	☐ Yes<br>☐ No<br>☐ NA
**SECTION 2: GDM KNOWLEDGE**	
Do you think that more pregnancies increase the chance of developing gestational diabetes? *	☐ Yes [1 point]<br>☐ No<br>☐ I do not know
Do you think that women who have had gestational diabetes have a greater chance of developing it again? *	☐ Yes [1 point]<br>☐ No<br>☐ I do not know
Do you think that weight gain/obesity before pregnancy may contribute to gestational diabetes? *	☐ Yes [1 point]<br>☐ No<br>☐ I do not
Do you think that a woman whose family member has diabetes has a higher chance of developing gestational diabetes? *	☐ Yes [1 point]<br>☐ No<br>☐ I do not know
Do you think that rapid weight gain	☐ Yes [1 point]

during pregnancy may contribute to gestational diabetes? *	☐ No<br>☐ I do not know
What is the usual time for diagnosing gestational diabetes in the absence of risk factors? *	☐ Week 12 – 22<br>☐ Week 24 – 28 [1 point]<br>☐ Week 32 – 35<br>☐ I do not know
What is the test (analysis) used to diagnose gestational diabetes? *	☐ Urine analysis<br>☐ Blood analysis<br>☐ Blood analysis after drinking glucose solution [1 point]<br>☐ I do not know
What is the treatment plan that is used for a woman with gestational diabetes? (You can choose more than one answer) *	☐ Diet and exercise [1 point]<br>☐ Insulin injection OR Oral diabetes medications [1 point]<br>☐ I do not know
Gestational diabetes...? (You can choose more than one answer) *	☐ Usually disappears after birth [1 point]<br>☐ Can affect the newborn baby if gestational diabetes is not treated [1 point]<br>☐ A woman with gestational diabetes may develop Type II diabetes in the future [1 point]<br>☐ I do not know
**SECTION 3: GDM KNOWLEDGE SOURCES**	
From where/whom did you hear about GDM? (You can choose more than one answer) *	☐ Hospital<br>☐ Hospital staff (specify) _________<br>☐ School<br>☐ Workplace<br>☐ TV<br>☐ Newspaper/ magazines<br>☐ Social media<br>☐ Other online resources<br>☐ Family/ friends<br>☐ Neighborhood<br>☐ Others (specify) _________<br>☐ NA

**Assessment of GDM Knowledge and Associated Factors among University Students in the UAE – A Cross-Sectional Study**	
Dear Respondent,<br>Please kindly fill the survey after signing the informed consent above. Thank you for your valuable time.<br>* Required	
القسم الأول: صفات وخصائص الحالة الاجتماعية	
* العمر:	☐ ________
* الجنس	☐ أنثى<br>☐ ذكر
*المنطقة:	☐ أبو ظبي<br>☐ دبي<br>☐ الشارقة<br>☐ عجمان<br>☐ رأس الخيمة<br>☐ أم القيوين<br>☐ الفجيرة
*المدينة:	☐ ________
الوزن:	☐ ________
الطول:	☐ ________
*الحالة الاجتماعية:	☐ أعزب/ عزباء<br>☐ متزوج/ متزوجة<br>☐ مطلق/ مطلقة<br>☐ أرمل/أرملة<br>☐ غيره ________
*هل كنتِ (أو زوجَتُك) حاملاً من قبل؟ (إذا كان السؤال مطابق لحالتك)	☐ لا<br>☐ إذا كانت الإجابة نعم، ________ فكم عدد حالات الحمل؟<br>☐ لا ينطبق
*اسم الكلية:	☐ كلية الإدارة والاقتصاد<br>☐ كلية العلوم الإنسانية والاجتماعية<br>☐ كلية تقنية المعلومات<br>☐ كلية الهندسة<br>☐ كلية التربية<br>☐ كلية القانون<br>☐ كلية الزراعة والطب البيطري<br>☐ كلية الطب والعلوم الصحية
القسم :	☐ ________
*البرنامج؟	☐ الجامعية<br>☐ دراسات عليا
*هل تعمل حالياً؟	☐ نعم<br>☐ لا

*إذا كنتَ تعمل حالياً، فأين تعمل؟	☐ اعمل في القطاع الصحي<br>☐ اعمل خارج القطاع الصحي<br>☐ لا ينطبق
*هل يوجد أحد في منزلك يعمل في المجال الصحي؟	☐ لا<br>☐ نعم__________
هل يوجد شخص في المنزل الذي تعيش فيه مصاب بمرض السكري؟ حدد*	☐ لا<br>☐ نعم__________
*هل انت مصاب بأمراض مزمنة؟	☐ لا<br>☐ نعم
*إذا كانت الإجابة نعم، (يمكنك اختيار أكثر من إجابة)	☐ مقدمات السكري<br>☐ داء السكري<br>☐ ارتفاع ضغط الدم<br>☐ ارتفاع الدهون/الكوليسترول<br>☐ أمراض القلب<br>☐ جلطة دماغية<br>☐ مرض الغدة الدرقية<br>☐ مرض السمنة<br>☐ غيرها__________<br>☐ لا ينطبق
* الحوامل)؟ هل سمعت من قبل بما يسمى (مرض السكري عند	☐ نعم<br>☐ لا
*هل تعرف أحد اصيب بمرض سكر الحمل؟	☐ نعم<br>☐ لا<br>☐ لا ينطبق
*هل أصبتِ (أو زوجَتُكَ) بسكري الحمل أثناء أي من حالات حملها؟	☐ نعم<br>☐ لا<br>☐ لا ينطبق
**القسم الثاني: المعرفة بمرض السكري عند الحوامل**	
*هل تعتقد ان زيادة مرات الحمل تزيد من فرصة الاصابة بسكر الحمل؟	☐ نعم [1 point]<br>☐ لا<br>☐ لا أعلم
هل تعتقد أن النساء الذين سبق لهم وأن أصيبوا بسك الحمل لديهم فرصة أكبر * للإصابة به مرة أخرى؟	☐ نعم [1 point]<br>☐ لا<br>☐ لا أعلم
هل تعتقد أن زيادة الوزن/ السمنة قبل الحمل قد يساهم في حدوث سكر الحمل؟*	☐ نعم [1 point]<br>☐ لا<br>☐ لا أعلم
هل تعتقد/ تعتقد أن المرأة التي أحد أفراد عائلتها مصاب بالسكري لديها فرصة أكبر للإصابة بسكر الحمل؟*	☐ نعم [1 point]<br>☐ لا<br>☐ لا أعلم
هل تعتقد أن سرعة زيادة الوزن أثناء الحمل قد يساهم في الاصابة بسكر * الحمل؟	☐ نعم [1 point]<br>☐ لا<br>☐ لا أعلم
ما هو الوقت المعتاد لتشخيص سكر الحمل في حال عدم وجود عوامل الخطر * المتعلقة بوجوده؟	☐ الاسبوع 12 -22<br>☐ الاسبوع 24 – 28 [1 point]<br>☐ الاسبوع 23 – 35

	☐ لا أعلم
*ما هو الاختبار (التحليل) المستخدم لتشخيص سكر الحمل؟	☐ تحليل البول<br>☐ تحليل الدم<br>☐ تحليل الدم بعد شرب محلول الجلوكوز [1 point]<br>☐ لا أعلم
ما هي الخطة العلاجية التي تستخدم للمصابة بسكر الحمل؟ (يمكن اختيار أكثر من إجابة) *	☐ الحمية والتمارين الرياضية [1 point]<br>☐ حقن الأنسولين OR<br>☐ أدوية السكر عن طريق الفم [1 point]<br>☐ لا أعلم
*سكر الحمل .....؟ (يمكن اختيار أكثر من إجابة)	☐ عادةً يختفي بعد الولادة [1 point]<br>☐ من الممكن أن يؤثر على الطفل المولود إذا لم يتم علاج سكر الحمل [1 point]<br>☐ من الممكن أن المرأة المصابة بسكر الحمل أن تصاب بداء السكري من النوع الثاني مستقبلاً [1 point]<br>☐ لا أعلم
*من أين سمعت (مصدر المعلومات) عن سكر الحمل عند الحوامل؟	☐ المستشفى<br>☐ المدرسة<br>☐ مكان العمل<br>☐ التلفزيون<br>☐ الجريدة / المجلة<br>☐ وسائل التواصل الاجتماعي<br>☐ مصادر أخرى من الإنترنت<br>☐ العائلة/ الأصدقاء<br>☐ الجيران<br>☐ غيرها. حدد__________

Institute of Public Health
College of Medicine and Health Sciences
United Arab Emirates University
P.O. Box 17666, Al Ain, UAE
معهد الصحة العامة
كلية الطب والعلوم الصحية
جامعة الإمارات العربية المتحدة
ص.ب 17666، العين، الإمارات العربية المتحدة
