# Supplementary material for: Gestational Diabetes Mellitus: A Cross-Sectional Survey of Its Knowledge and Associated Factors among United Arab Emirates University Students
Source: Int J Environ Res Public Health. 2022 Jul 8;19(14):8381. doi: 10.3390/ijerph19148381 (PMC9321366; doi:10.3390/ijerph19148381)
Supplement: Supplementary file 1 [file ijerph-19-08381-s001.zip › Supplementary Table S1 - missing data_GDM knowledge study.pdf]

Supplementary table S1 showing percentage missing data for the 735 eligible students

Variables	N* = 735 (%missing)
Age(years)	735 (0%)
Gender	735 (0%)
Program	735 (0%)
Marital status	735 (0%)
Working status	735 (0%)
Anyone in your home working in the Health Sector	735 (0%)
Family history of diabetes	735 (0%)
Weight(kg) (among 735 total)	698 (5.0%)
Height(m) (among 735 total)	684 (6.9%)
BMI (kg/m^2) (among 735 total)	677 (7.9%)
Outcome (ever heard and never heard of GDM)	735 (0%)

***Total number of participants who responded to a question**

Missing data for 480 students included in the GDM knowledge level analysis

Variables	N* = 480 (%missing)
Age(years)	480 (0%)
Gender	480 (0%)
Program	480 (0%)
Marital status	480 (0%)
Working status	480 (0%)
Anyone in your home working in the Health Sector	480 (0%)
Family history of diabetes	480 (0%)
Knowing someone who had GDM	480 (0%)
Weight(kg) (among 480 total)	457 (4.8%)
Height(m) (among 480 total)	452 (5.8%)
BMI (kg/m^2) (among 480 total)	449 (6.5%)
Outcome (poor, fair, good)	480 (0%)

***Total number of participants who responded to a question**
