# Supplementary material for: Gestational Diabetes Mellitus: A Cross-Sectional Survey of Its Knowledge and Associated Factors among United Arab Emirates University Students
Source: Int J Environ Res Public Health. 2022 Jul 8;19(14):8381. doi: 10.3390/ijerph19148381 (PMC9321366; doi:10.3390/ijerph19148381)
Supplement: Supplementary file 1 [file ijerph-19-08381-s001.zip › Supplementary Table S2 - Sociodemographic characteristics of 902 eligible students.pdf]

Supplementary Table S2. Sociodemographic characteristics of 735 eligible students and comparisons between those who "Ever heard of GDM" and those who "Never heard of GDM"

Sociodemographic Characteristics	Total population N*	n (%) / Median (IQR)	Ever heard of GDM n=535 (72.8%)	Never heard of GDM n=200 (27.2%)	Pvalue[a]
**Age(years)**	735	21.0 (3.0)	21.0 (3.0)	20.0 (3.0)	0.797
**Weight (kg)**	698	61.0 (21.4)	60.0 (20.0)	64.0 (21.0)	0.020
**Height (m)**	684	1.60 (0.09)	1.60 (0.09)	1.63 (0.14)	<0.001
**BMI (kg/m^2)**	677	23.3 (6.5)	23.1 (6.9)	23.5 (6.0)	0.966
**Gender**	735				
**Male**		155 (21.1)	73 (47.1)	82 (52.9)	
**Female**		580 (78.9)	462 (79.7)	118 (20.3)	<0.001
**Program**	735				
**Undergraduate**		605 (82.3)	441 (72.9)	164 (27.1)	
**Postgraduate**		130 (17.7)	94 (72.3)	36 (27.7)	0.892
**Marital status**	735				
**Single**		629 (85.6)	450 (71.5)	179 (28.5)	
**Married**		106 (14.4)	85 (80.2)	21 (19.8)	0.064
**Working status**	735				
**Yes**		89 (12.1)	61 (68.5)	28 (31.5)	
**No**		646 (87.9)	474 (73.4)	172 (26.6)	0.337
**Anyone in your home working in the Health Sector**	735				
**Yes**		140 (19.1)	100 (71.4)	40 (28.6)	
**No**		595 (80.9)	435 (73.1)	160 (26.9)	0.688
**Family history of diabetes**	735				
**Yes**		369 (50.2)	283 (76.7)	86 (23.3)	
**No**		366 (49.8)	252 (68.9)	114 (31.2)	0.017
**BMI category**	677				
**Normal weight**		339 (50.1)	251 (74.0)	88 (26.0)	
**Underweight**		96 (14.2)	72 (75.0)	24 (25.0)	
**Overweight**		153 (22.6)	107 (69.9)	46 (30.1)	
**Obesity**		89 (13.1)	69 (77.5)	20 (22.5)	0.594

Data were expressed as median (IQR- Interquartile range) or n (%) unless stated otherwise. *=Total number of eligible students who responded to a particular question. [a]=Chi square used for categorical variables and Mann-Whitney U test for continuous variables.
