# Supplementary material for: Gestational Diabetes Mellitus: A Cross-Sectional Survey of Its Knowledge and Associated Factors among United Arab Emirates University Students
Source: Int J Environ Res Public Health. 2022 Jul 8;19(14):8381. doi: 10.3390/ijerph19148381 (PMC9321366; doi:10.3390/ijerph19148381)
Supplement: Supplementary file 1 [file ijerph-19-08381-s001.zip › Supplementary Table S3 - GDM knowledge points frequency.pdf]

Supplementary Table S3 showing frequency of GDM Knowledge points (0-12) among study population (480 Students)

Total GDM Knowledge Points	Freq.	Percent	Cum.
**0**	7	1.46	1.46
**1**	5	1.04	2.50
**2**	18	3.75	6.25
**3**	25	5.21	11.46
**4**	60	12.50	23.96
**5**	59	12.29	36.25
**6**	76	15.83	52.08
**7**	82	17.08	69.17
**8**	64	13.33	82.50
**9**	40	8.33	90.83
**10**	25	5.21	96.04
**11**	14	2.92	98.96
**12**	5	1.04	100.00
**Total**	**480**	**100.00**	
