# Supplementary material for: Gestational Diabetes Mellitus: A Cross-Sectional Survey of Its Knowledge and Associated Factors among United Arab Emirates University Students
Source: Int J Environ Res Public Health. 2022 Jul 8;19(14):8381. doi: 10.3390/ijerph19148381 (PMC9321366; doi:10.3390/ijerph19148381)
Supplement: Supplementary file 1 [file ijerph-19-08381-s001.zip › Supplementary Table S4 - regression analysis of GDM knowledge status for 902 eligible students.pdf]

Supplementary Table S4. Simple and multiple regression analysis between GDM knowledge status (ever heard of GDM and never heard of GDM) and students' characteristics (N=735)

Sociodemographic Characteristics		Unadjusted* OR (95% CI)	Adjusted OR (95% CI) *
**Gender**	Male	ref	
	Female	4.398 (3.024-6.397) [a]	3.444 (1.944-6.102) [a]
**Family history of diabetes**	No	ref	
	Yes	1.489 (1.073-2.065) [a]	1.311 (0.913-1.882)
**Weight (kg)**		0.989 (0.980-0.999) [a]	1.010 (0.997-1.023)
**Height (m)**		0.002 (0.000-0.019) [a]	0.079 (0.003-2.270)

***=Ordinal logistic regression used. [a]=statistically significant after regression ($p<0.05$).**
