# Supplementary figures and images for: Gestational Diabetes Mellitus: A Cross-Sectional Survey of Its Knowledge and Associated Factors among United Arab Emirates University Students
Source: Int J Environ Res Public Health. 2022 Jul 8;19(14):8381. doi: 10.3390/ijerph19148381 (PMC9321366; doi:10.3390/ijerph19148381)

## Supplementary Figure S2 showing study population

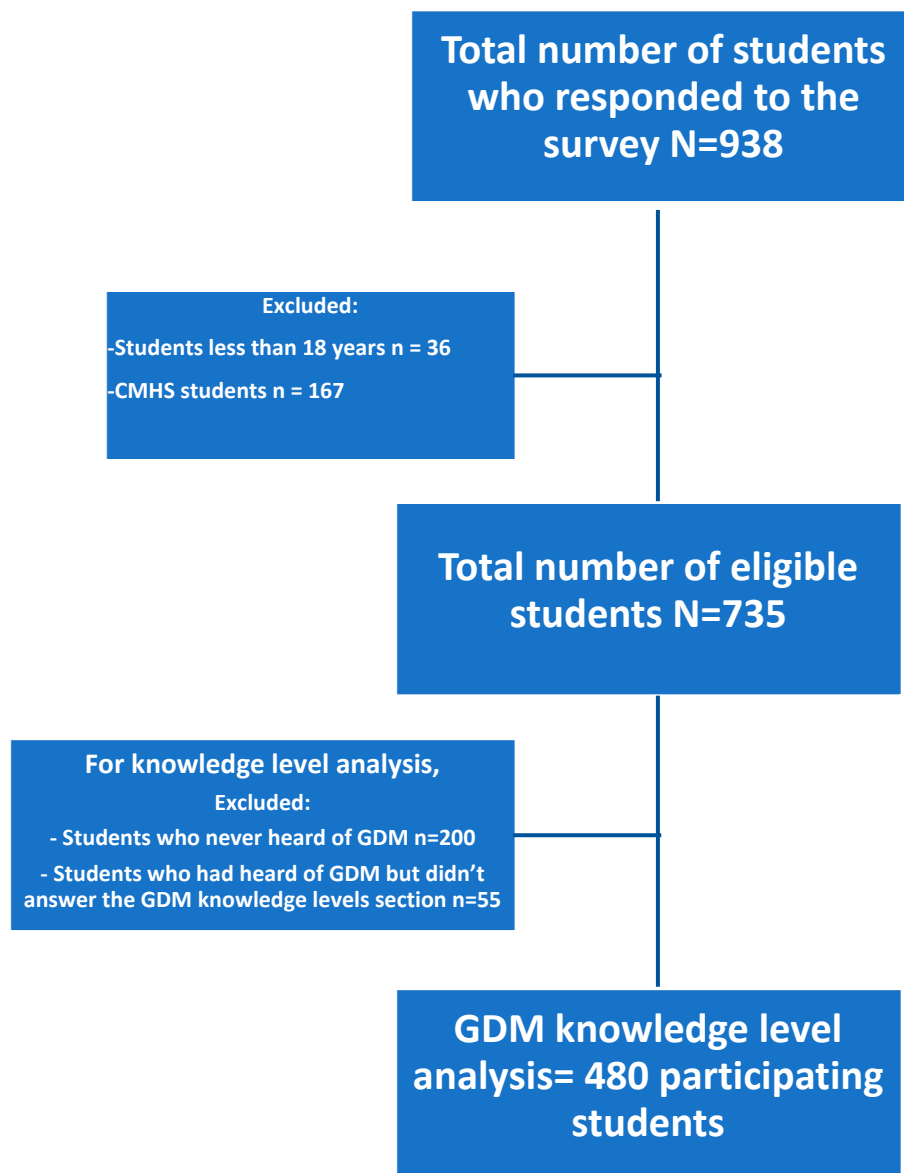

Supplement: Supplementary file 1 [file ijerph-19-08381-s001.zip › Supplementary Figure S2 - Study population -GDM knowledge study.pdf]
